# Dr. James H. Hysell

**Published:** 1905-07

**Authors:** 


Dr. James H. Hysell, University of Buffalo Medical Department, 1861, died at his home in Pomeroy, O., May 10, 1905, aged 67 years. He was assistant surgeon of the ninth Virginia volunteer infantry, chief surgeon of the second division first army corps, chief surgeon of Cienfuegos province and later medical disbursing officer at Santiago, during the war with Spain. He was also surgeon of the first West Virginia veteran infantry during the civil war.

